# SEM-Based Analysis of Carbon Emission Reduction Pathway Study during the Materialization Stage of Prefabricated Buildings: Evidence from Shenyang and Guiyang, China

**DOI:** 10.1155/2022/9721446

**Published:** 2022-09-19

**Authors:** Rui Zhu, Lihong Li

**Affiliations:** School of Management, Shenyang Jianzhu University, Shenyang 110168, China

## Abstract

In recent years, in the process of promoting prefabricated buildings, problems such as waste of resources and energy have been present, which have seriously hindered the realization of carbon emission reduction benefits of prefabricated buildings. Especially during the materialization stage of prefabricated buildings which involves the most engineering activities and the most extensive sources of carbon emissions, it is urgent to further meet the low-carbon development of the construction industry. This study takes the 4 substages of design and development, component production, transportation, and installation during the materialization stage as the point of penetration and identifies the carbon reduction impact pathways based on the 3E (Environment-Economy-Energy) system theory in 5 dimensions: government policy, management mode, technology level, economy input, and energy structure. The data are collected through the questionnaire survey, and structural equation modeling (SEM) is utilized to examine the hypothesis and impact dimensions of the study. The results confirm that the management mode has the strongest effects on carbon emission reduction, followed by government policy, economy input, and technology level, and the energy structure has the weakest effects. This study presents the key carbon reduction pathways during the materialization stage of prefabricated buildings and provides recommendations for different participants to optimize policy guidance, strengthen management supervision, accelerate technology research and development, increase economy input, and optimize energy structure, with a view to achieving low-carbon governance capacity, management mode, technology system, capital, and energy utilization, and also enriches the theory in the field of prefabricated buildings carbon emission reduction, which can better achieve low-carbon development of prefabricated buildings.

## 1. Introduction

Carbon dioxide and other greenhouse gas emissions are gradually increasing as human society becomes more urbanized, and figuring out how to limit carbon emissions has become a significant challenge that all of humanity urgently needs to tackle. The construction industry sector consumes a high proportion of energy [1] and emits a significant amount of greenhouse emissions around the world [2]. As a result, reducing carbon emissions in the construction industry is critical. Many countries, including China, have backed prefabricated buildings in recent years because the economic, environmental, and social benefits can be provided in meeting human society's development needs [3]. The ability of prefabricated buildings to reduce carbon emissions in the construction industry has been demonstrated [4]. However, compared with most western countries, the use of prefabricated buildings in China is still at the developmental stage, and theoretical research on the low-carbon development of prefabricated buildings is also lacking [5], resulting in the benefits of all aspects not being fully utilized, mainly including the lack of policies and regulations, barriers to the application of technology, weak competition, low management level, and other issues [6]. The low carbon development of prefabricated buildings is influenced by these problems to different extents.

In 2020, at the 75th United Nations Congress, the Chinese government announced to the world that it would strive to achieve carbon peaking by 2030 and carbon neutrality by 2060. In the context of carbon peaking and carbon neutrality goals, China's construction industry urgently needs a low-carbon transformation and the low-carbon development of prefabricated buildings has ushered in new opportunities. It is worth noting that the materialization stage of prefabricated buildings involves a wide variety of carbon emission sources, and the carbon emission reduction paths are difficult to effectively achieve carbon emission reduction effects [7]. Achieving carbon emission reduction during the materialization stage of prefabricated buildings cannot only solve the obstacles to the low-carbon development of the construction industry from the root but also improve the theories related to the low-carbon development of the construction industry and further reduce the impact of construction industry development on the environment and improve the human living environment. Therefore, many studies have been conducted in academia to address these issues. So far, the academic discussion on these issues can be roughly divided into three categories. Most of the studies on carbon emission reduction during the materialization stage were based on the establishment of the BIM of carbon emission, by understanding the specific carbon emissions of various engineering activities during the materialization stage and then implementing carbon emission control [8, 9]. Some other scholars conducted the carbon emission study through quantitative methods, used the carbon footprint measurement method to determine the accounting scope and types of carbon emissions, and put forward countermeasures and suggestions for carbon emission reduction [10]. There are also studies that show that carbon emissions from prefabricated buildings vary with the prefabrication rate and that carbon emissions from prefabricated buildings decrease slightly as the prefabrication rate increases, with suggestions for improving carbon emission reduction from the prefabrication process [11, 12]. However, these methods do not take into account the effects of carbon emission reduction of all participants involved during the materialization stage of prefabricated buildings, and the specific conditions of different regions are different, so the conclusions obtained do not meet the needs of all parties involved and have certain limitations.

In this study, considering that carbon emission reduction during the materialization stage of prefabricated buildings is a system with complex influencing factors and the emission reduction role of all participants involved cannot be ignored. The study adopts a multivariate data analysis method to study carbon emission reduction during the materialization stage of prefabricated buildings, identifying carbon emission reduction pathways based on the 3E system theory, obtaining data through the questionnaire survey, and applying SEM for analysis. The study also clarifies the key carbon emission reduction paths and provides suggestions for the implementation of low-carbon measures for all participants involved. Besides, the study improves the theory of low-carbon development and the quality of people's living environment and enriches the application of SEM in the field of low-carbon development of prefabricated buildings.

## 2. Literature Review

In this study, the materialization stage refers to the process of prefabricated buildings from nonexistence to passing into existence. According to the sequence of engineering activities with the participants of the materialization stage, it is determined that the materialization stage includes four subphases: design and development, component production, transportation, and installation stage [8]. Some scholars have compared carbon emissions during the materialization stage of prefabricated buildings with traditional cast-in-place buildings and found that the materialization stage has obvious benefits in terms of carbon emission reduction; during the production stage of the materialization stage, carbon emission is relatively high [13–15]. However, some scholars have found that the process of controlling carbon emissions during the materialization stage is often difficult and complex because the different substages involved produce different carbon emissions and channels [16, 17]. It is difficult to implement effective carbon emission reduction paths for the participants. Therefore, this study selects the carbon reduction paths of the materialization stage of prefabricated buildings as the research object, aiming to reduce the carbon emission of the materialization stage of prefabricated buildings.

This study has very significant implications for the further development of prefabricated buildings. Based on previous studies, it is clear that many factors influence the carbon emissions of the materialization stage of prefabricated buildings. Sandanayake et al. [18] have quantified the direct and indirect carbon emissions of the materialization stage of prefabricated buildings and compared them with traditional cast-in-place buildings to identify technical, management, and policy factors. Luna-Tintos et al. [19] have suggested achieving low carbon of prefabricated buildings in terms of advanced technology, personnel management, and optimization of the energy structure. Xue et al. [20] have pointed out that technological upgrading and increasing economy input are the keys to realizing green and low-carbon buildings through research on building stakeholders. Many scholars have put forward the influencing factors during the materialization stage of prefabricated buildings from different perspectives, but these influencing factors are not systematic and comprehensive enough. In the theory of low carbon development, the 3E system theory points out that economy, energy, and environment form an interrelated and contradictory ternary system, and none of them can be ignored. Ma et al. [21] have used the 3E system theory to study the ecological efficiency of the Yangtze River Delta in China and made corresponding suggestions to achieve low-carbon and environmental protection. Therefore, the study combines the 3E system theory with existing studies and focuses on 5 influential dimensions of the materialization stage of prefabricated buildings: government policy, management mode, economy input, technology level, and energy structure. Some studies have found that the government should give full play to its guiding role to successfully realize low-carbon policies while safeguarding the participants' interests through the implementation of policies such as tax incentives and financial subsidies [22–24]. Management is carried out throughout the materialization stage. Some studies have pointed out that participants' low-carbon management of the resources and energy at their disposal can achieve carbon emission reductions [25, 26]. For example, the low-carbon management of machinery and equipment by component manufacturers can reduce energy consumption and prolong the service life of the equipment [17]. Other studies have found that adequate capital is one of the indispensable elements to achieving carbon emission reduction, and the rational use of capital for upgrading machinery and equipment and technological energy research is very important to achieve carbon emission reduction [20, 27]. In terms of carbon emission reduction through technology, some studies have shown that the use of 3D printing, the Internet of things (IoT), and other technologies in the process of component production and installation cannot only reduce the cost but also reduce the uncertainty of component assembly, thus reducing waste [6, 17]. During the materialization stage, there is huge energy demand. By establishing renewable energy systems as power sources, such as solar photovoltaic (PV) systems, the dependence on traditional fossil fuels is reduced, and carbon emissions are directly and effectively reduced [28, 29].

In terms of carbon emission reduction study methods for the materialization stage of prefabricated buildings, SEM can effectively reveal the critical impact paths and the relationships between the impact paths through hypothesis models [30]. In the field of low-carbon agriculture, J. Hou and B. Hou [31] focused on how different factors affect the low-carbon development of China's agriculture based on SEM and determined the key influencing paths to provide a reference for the formulation of low-carbon agricultural support policies. In terms of low-carbon travel, Yin and Shi [32] used SEM to analyze the main influencing factors of households' low-carbon behavior and proposed different targeted policy recommendations. To achieve carbon emission reduction of prefabricated buildings, Du et al. [33] have established the SEM model from the perspective of the supply chain to determine the key paths affecting carbon emission of prefabricated building supply chains from government, technology, and more and put forward corresponding suggestions. In summary, SEM has been widely used in agriculture and other fields as a method of carbon emission reduction path analysis, and SEM has the advantages of simultaneously analyzing multiple factors, determining the key factors that affect the target subject, and allowing the measurement error of each variable [34]. Therefore, this study can use SEM as the study tool for the analysis of carbon reduction paths during the materialization stage of prefabricated buildings, which cannot only analyze multiple carbon reduction pathways simultaneously but also fully explore the key carbon reduction paths that have a significant impact on the research object.

## 3. Materials and Methods

**Figure 1:**
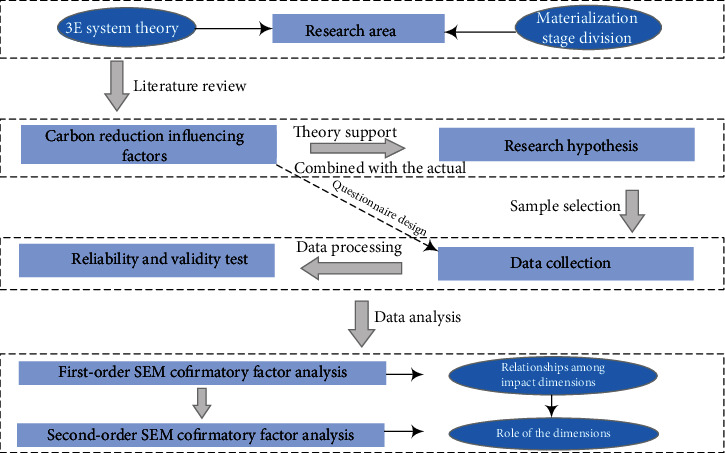
Logic diagram of research methods.

The entire process as well as the logical relationship between methods adopted in this study is shown in Figure 1. The introduction of this section on the study materials and methods mainly includes six steps; the first step is to determine the area of the study. The second step is to determine the list of influencing factors of carbon emission reduction based on the literature review. The third step is to put forward the path hypothesis based on previous studies. The fourth step is to collect the data according to the carbon reduction influencing factors list. The fifth step is to test the reliability and validity of the data and, finally, apply SEM for data analysis.

### 3.1. Research Area

**Figure 2:**
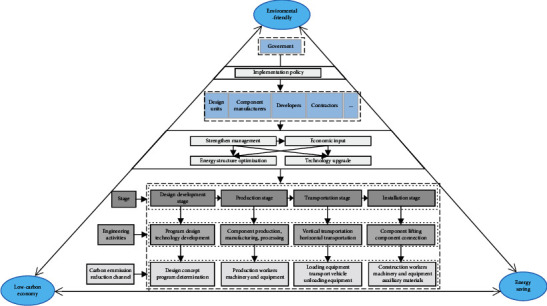
Scoping of carbon emission reduction during the materialization stage of prefabricated buildings based on 3E.

This study analyzes carbon emission reduction paths during the materialization stage of prefabricated buildings, including four substages: design and development, component production, transportation, and installation. However, there are many carbon emission reduction pathways in each substage, and the impact aspects are complicated, so it is difficult to define the scope of the analysis. Based on the 3E system theory [35], it is known that there is a mutually influencing and constraining relationship between environment, energy, and economy in low-carbon development. The purpose of this study is to achieve energy saving and emission reduction in the construction industry as well as to minimize its impact on the environment and promote the development of a low carbon economy. Therefore, the study defines the scope of carbon emission reduction paths during the materialization stage of prefabricated buildings based on the research of previous scholars and the 3E system theory (see Figure 2). Firstly, the government plays a leading role in the upstream of the model. By introducing relevant low-carbon policies, the government urges component manufacturers, design units, and contractors, which are in the middle and lower reaches of the model, to take corresponding low-carbon measures. Secondly, the model reveals the engineering activities and sources of carbon emissions during each substage and determines the scope of carbon emission reduction. Finally, in the model, all participants work together to achieve the goals of environment friendliness, low-carbon economy, and energy saving through corresponding low-carbon measures.

### 3.2. Identification of Influencing Factors of Carbon Emission Reduction during the Materialization Stage of Prefabricated Buildings

After determining the study area, the study further identifies the influencing factors of carbon reduction and determines the list of influencing factors of carbon reduction during the materialization stage of prefabricated buildings by summarizing and organizing the relevant studies of previous scholars (see Table 1).

### 3.3. Research Hypothesis

In the study, it is found that there may be complex interactions between influencing factors of carbon emission reduction in each dimension of the materialization stage of prefabricated buildings. Therefore, on the basis of understanding the actual situation of carbon emission from prefabricated buildings in China and literature review, this study proposes the following hypothesis.

#### 3.3.1. Government Policy

Through the implementation of policies, the government leads prefabricated building-related enterprises to achieve the purpose of carbon emission reduction and has a certain incentive effect on enterprises, directly promoting enterprises to adopt low-carbon measures [6, 15, 18].

H1: “Government policy” has a positive impact on “management mode.”

H2: “Government policy” has a positive impact on “economy input.”

H3: “Government policy” has a positive impact on “energy structure.”

H4: “Government policy” has a positive impact on “technology level.”

H5: “Government policy” has a positive impact on “carbon emission reduction during the materialization stage of prefabricated buildings.”

#### 3.3.2. Management Mode

Prefabricated building-related enterprises can reduce the consumption of energy resources by improving the management of personnel, materials, machinery, and equipment. Improving the low-carbon management mode requires upgrading the relevant technology and equipment and increasing economic input [15, 16].

H6: “Management mode” has a positive impact on “economy input.”

H7: “Management mode” has a positive impact on “energy structure.”

H8: “Management mode” has a positive impact on “technology level.”

H9: “Management mode” has a positive impact on “carbon emission reduction during the materialization stage of prefabricated buildings.”

#### 3.3.3. Economy Input

Adequate funding is an important guarantee for low-carbon technology and new energy research and development. Reducing carbon emissions during the materialization stage of prefabricated buildings also requires a lot of financial support [5, 16, 26].

H10: “Economy input” has a positive impact on “technology level.”

H11: “Economy input” has a positive impact on “energy structure.”

H12: “Economy input” has a positive impact on “carbon emission reduction during the materialization stage of prefabricated buildings.”

#### 3.3.4. Energy Structure

The use of clean energy such as solar energy during the materialization stage of prefabricated buildings can effectively reduce carbon dioxide emissions and has a low environmental impact and low pollution [25, 29].

H13: “Energy structure” has a positive impact on “carbon emission reduction during the materialization stage of prefabricated buildings.”

#### 3.3.5. Technology Level

Low-carbon technology is the core driver of low-carbon development in prefabricated buildings, and the higher the level and popularity of low-carbon technology in the materialization stage of prefabricated buildings, the stronger the carbon reduction ability [2, 3].

H14: “Technology level” has a positive impact on “carbon emission reduction during the materialization stage of prefabricated buildings.”

### 3.4. Data Collection

This study collected the data through a questionnaire survey. The questionnaire survey was mainly distributed to the prefabricated building practitioners in Shenyang and Guiyang, China. Shenyang is one of the first national demonstration cities of prefabricated buildings and the central city of the old industrial base in Northeast China; the local government attaches great importance to the development of the prefabricated building industry and has formed a prefabricated building development model in line with local characteristics. However, Shenyang's building carbon emission is always high, and relying on prefabricated buildings to reduce Shenyang's building carbon emission is of great significance. Guiyang is a city with the rapid development of prefabricated buildings in south China and has sufficient technical funds. However, there are still some deficiencies in low-carbon policy guidance for prefabricated buildings. The reason for choosing Shenyang and Guiyang is that both cities are vigorously developing prefabricated buildings with outstanding achievements, and the data obtained are representative to a certain extent.

The questionnaire is composed of two parts. The first part consists of the basic information of respondents, including gender, educational background, nature of work unit, and working experience in the prefabricated building industry. The second part contains 23 questions, which are designed based on the carbon reduction paths of the five dimensions in Table 1. A 5-point Likert scale was used to solicit respondents' attitudes toward the measurement program. Questionnaires were distributed to component manufacturers, developers, contractors, design units, supervisory units, scientific research institutions, and universities, etc. A total of 240 questionnaires were distributed in the study, and 220 questionnaires were effectively recovered by removing the indiscriminate and omitted questionnaires, with an effective recovery rate of 91.67%. The survey results are shown in Table 2.

### 3.5. Data Processing

Before forming the official scale, this study conducted consultation interviews with relevant experts and representative practitioners, summarized and modified the original questionnaire by combining relevant opinions, and further improved the scale. Whether the questionnaire data in the study can be used for further analysis of SEM needs to be tested for reliability and validity to ensure that they meet the factor analysis criteria. As shown in Table 3, in the reliability test, Cronbach's alpha for the latent variables ranged between 0.823 and 0.931 and is greater than 0.8. Corrected Item Total Correlation (CITC) is also used to evaluate the convergence of the scale. The results showed that CITC values of each item and the whole were above the recommended value of 0.4. In the validity test, in this study, Kaiser-Meyer-Olkin (KMO) value and Bartlett's test are selected as validation indicators. The KMO value of the latent variables is greater than 0.7, and the *P* value of the Bartlett test of potential variables is lower than 0.000. Therefore, the sample had good reliability and validity.

### 3.6. Data Analysis

After the reliability and validity of the questionnaire data met the criteria, the confirmatory factor analysis (CFA) of SEM was applied to the collected data in this study using Amos 24.0. The aim of conducting CFA is to verify the path hypothesis proposed in this study. To evaluate the model performance, the study refers to several well-established indicators. In terms of fitness, the study selected the chi-square/degree of freedom (*χ*^2^/df), the root-mean-squared error of approximation (RMSEA), the root-mean-square residual (RMR), the comparative fit index (CFI), the incremental fit index (IFI), the Tucker-Lewis index (TLI), the parsimonious goodness-of-fit index (PGFI), and the parsimonious normed fit index (PNFI). Model validity is evaluated by the critical ratio (CR). Acceptable values for the indicator are recommended as |CR| > 1.96. Relationships among different variables are interpreted based on the standardized coefficients.

## 4. Results

### 4.1. First-Order SEM Confirmatory Factor Analysis

**Figure 3:**
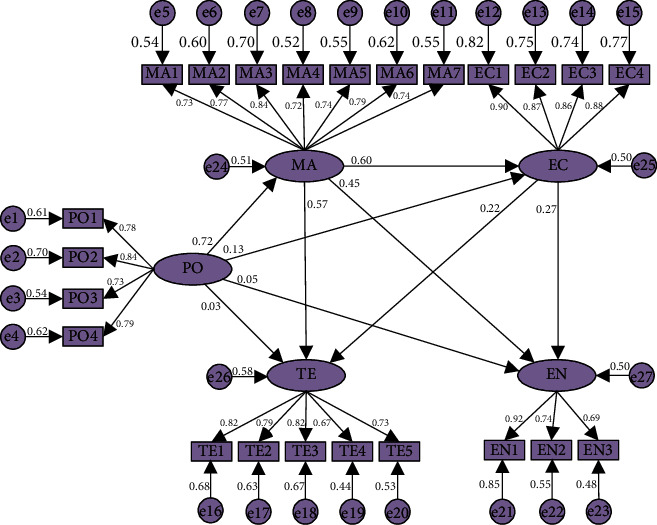
Standardized factors loadings and path coefficients of the first-order SEM.

To initially verify the path hypothesis in this study, the study conducted a first-order SEM confirmatory factor analysis of the relationship between latent variables. The survey data were imported into Amos24.0 software for calculation, and the first-order model was established as shown in Figure 3.

The analysis shows that there is no negative error term in the first-order SEM and the standardized coefficients are in the range of 0.67-0.92, which is in line with the standard range of 0.5-0.95. The model fitness indicators are shown in Table 4, which are all within the acceptable range, and there is no violation of estimation. The study indicates that the first-order model of carbon emission reduction paths during the materialization stage of prefabricated buildings fits well and has a high degree of fit, and the model establishment is scientific and reasonable.

The standardized path coefficients of the first-order SEM are shown in Table 5. The C.R. of the path hypothesis in the table are greater than 1.96 except for the H2, H3, and H4, and all indicators reach a significant level of 0.05, indicating that there is a high correlation between the latent variables and there may be another higher-order common factor influencing them. Therefore, the second-order model can be considered for further analysis.

### 4.2. Second-Order SEM Confirmatory Factor Analysis

**Figure 4:**
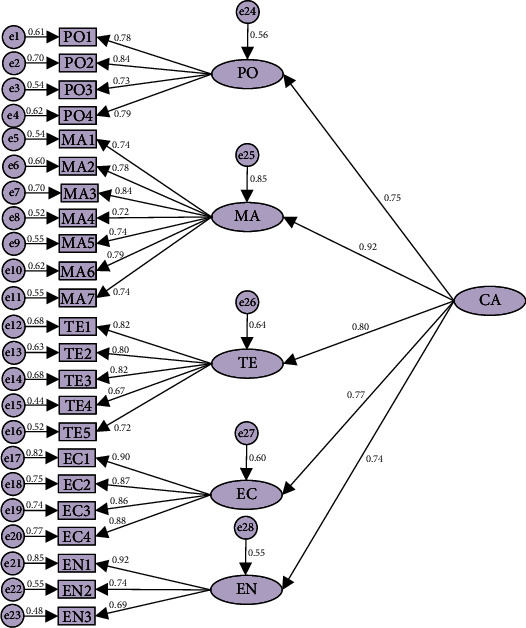
Standardized factor loadings and path coefficients of the second-order SEM.

According to the above analysis, the first-order SEM can be well adapted to the data, and there is a medium to high correlation among the latent variables, so it can be assumed that there is a common latent factor of higher order. Combining the results of questionnaire design and factor analysis, this higher-order potential factor is named carbon emission reduction during the materialization stage of prefabricated buildings (CA), and the second-order SEM is drawn accordingly, as shown in Figure 4.

The values of each index in the second-order SEM are within the acceptable range, and the model fitness indexes and standardized path coefficients are shown in Tables 6 and 7. Through the analysis of the model indexes and standardized path coefficients, the values of each index are within the acceptable range, and the C.R. are all greater than 2.58, and all of them reach the 0.001 significance level, showing a strong significance and a good fit. Therefore, the constructed second-order SEM of the carbon emission reduction path during the materialization stage of prefabricated buildings performs relatively well.

## 5. Discussion

### 5.1. First-Order SEM Result Analysis

**Figure 5:**
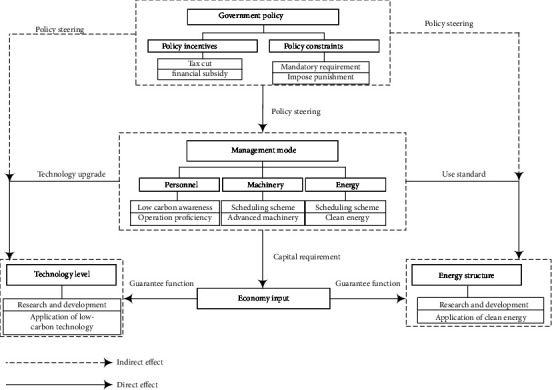
The influence between carbon emission reduction dimensions.

The discussion of the first-order SEM of carbon emission reduction paths during the materialization stage of prefabricated buildings is shown in Figure 5.

“Government policy” has a significant effect on “management mode” with a positive correlation, indicating that the implementation of relevant government low-carbon policies will promote the optimization of the low-carbon management mode of prefabricated building-related enterprises (H1). This is the same as the effect of policy-led carbon reduction in previous studies [22, 24]. Therefore, it is necessary for the government to carefully consider and formulate policies with mandatory and incentive. On the one hand, the government must bind participants to fulfill their low-carbon obligations. On the other hand, the government should provide incentives such as policy preferences to the participants of low-carbon measures. In addition, compared with previous studies [3, 23], this study also finds some special features that H2, H3, and H4, which are rejected in this study, indicate that the effects of “government policy” on “economy input,” “energy structure,” and “technology level” are not significant. Because the influence of “government policy” on the above three parties is mainly indirectly generated by guiding participants to improve the management mode, the direct effect is not significant, which also indicates that there is a shortage of government policy promotion and guidance in these three aspects, and the relevant policies should be improved as soon as possible to meet the requirements of low-carbon development.

“Management mode” has a high degree of influence on “economy input,” “energy structure,” and “technology level,” with significant correlation (H6, H7, and H8). The realization of low-carbon management during the materialization stage of prefabricated buildings can be divided into three aspects of management: personnel, energy, and machinery management [19, 26]. In terms of personnel management, low-carbon management requires the development of personnel's low-carbon awareness and operational proficiency in advanced low-carbon technologies and equipment. Therefore, improving the management mode will promote the input of capital and the technology level. In terms of energy management, the production, transportation, and installation stages of components involve the use of a variety of energy sources, and the low-carbon management of energy mainly lies in the formulation of reasonable energy use standards and optimization of energy structure. In terms of machinery management, a reasonable mechanical equipment scheduling program can reduce unnecessary waste and shorten the time cost.

The impact of “economy input” on “technology level” and “energy structure” indicates that prefabricated building-related enterprises can promote the upgrading and research of low-carbon technologies and low-carbon energy during the materialization stage by investing more funds (H10, H11). Reducing carbon emissions by applying low-carbon advanced technologies and introducing efficient and clean energy sources are extremely critical approaches [4, 20]. This study finds through comparison that in the past the participants focused more on low cost and high return. However, as low-carbon development continues, more and more participants realize that low pollution and high environmental protection are imperative. In order to achieve a low-carbon transition, all relevant participants have started to increase their economy input into low-carbon technologies and clean energy research and development. This results in increased costs in the short term. However, in the long run, the economic, environmental, and social benefits of achieving low-carbon goals are enormous. This study finds that “economy input” has a lower impact on “technology level” and “energy structure” than “management mode.” While there is no doubt that financial security promotes low-carbon technology and energy R&D, the direct impact of the “management mode” and the indirect impact of “government policy” not only contribute to “economy input” but also have a more comprehensive impact on technology upgrading and energy mix optimization.

### 5.2. Second-Order SEM Result Analysis

**Figure 6:**
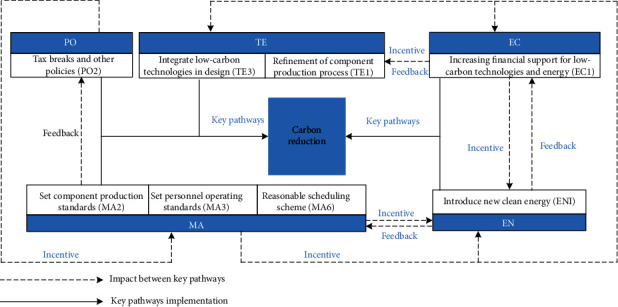
Determine the key carbon emission reduction path in the physical and chemical stage of prefabricated buildings.

A second-order SEM analysis of the carbon reduction paths during the materialization stage of prefabricated buildings is shown in Figure 6.

The degrees of influence of “government policy,” “management mode,” “technology level,” “economy input,” and “energy structure” on carbon emission reduction during the materialization stage of prefabricated buildings are 0.75, 0.92, 0.80, 0.77, and 0.74, respectively, which are more than 0.5, indicating that the carbon emission reduction paths of the five dimensions have significant effects on carbon emission reduction during the materialization stage of prefabricated buildings (H5, H9, H12, H13, and H14). The implementation effects of the above five dimensions of carbon reduction pathways are not the same. Guided by “government policy,” all participants improve the “management mode,” which in turn affects the “technology level,” “economic input,” and “energy structure.” Therefore, “management mode” plays a top-down role and has the most significant carbon reduction effect [6, 18]. In this study, it is important to pay special attention to the optimization of “energy structure” during the materialization stage which is realized under the joint action of the other four dimensions. Therefore, the influence of “energy structure” on carbon emission reduction is the weakest. This study also found through comparative analysis that the observed variables of the same dimension in the second-order SEM have larger normalized path coefficients in the production and installation phases compared with the design development and transportation phases. The production stage, where various raw materials are made into the required components through production and processing, is the key aspect that distinguishes prefabricated buildings from traditional cast-in-place buildings. The component installation stage is the combination of components by lifting and connecting them, and some building auxiliary materials are also required. These two substages involve a large number of engineering activities and consume more resources and energy. Therefore, participants should focus on implementing low-carbon measures in the component production and installation phases.

Based on the determination of the degree of influence of the five carbon reduction dimensions, this study identifies the critical pathways for carbon reduction in each dimension. In the “government policy” dimension, it can be found that the policy support given to component manufacturers that meet carbon emission standards has the greatest impact (PO2). Since component manufacturers are the main implements of carbon emission reduction measures during the production stage, the incentive and constraining effects of government policies make the components produced by component manufacturers meet low-carbon requirements, which is conducive to the healthy development of prefabricated buildings. Since “management mode” has the greatest impact on carbon emission reduction, it is crucial to set standards for component production workers and propose reasonable solutions for resource and energy scheduling at the installation site (MA2, MA3, and MA6). Prefabricated building-related enterprises train their employees through reasonable management methods, which not only improves the technical level of personnel and ensures the quality of work but also makes the low-carbon concept more deeply rooted. Meanwhile, the materialization stage involves many workers, materials, and machinery, and the participants should realize the reasonable allocation of internal resources, communicate information with other stakeholders on time, and coordinate internal and external relations through reasonable management methods to ensure the smooth implementation of carbon emission reduction. On the “technology level,” the integration of low-carbon technologies into the design has the greatest impact on carbon emission reduction (TE1, TE3). In order to reduce carbon emission during the materialization phase of prefabricated buildings, low-carbon technologies are gradually beginning to receive attention. In the context of coordinated development of intelligent construction and building industrialization, various digital technologies such as BIM, IoT, and big data have been widely used. Under such circumstances, there is an urgent need to accelerate the progress of low-carbon technologies to achieve low-carbon development. In terms of “energy structure,” with the concept of low carbon gaining more and more attention from all walks of life, clean energy is gradually occupying an increasingly important position due to its low carbon, high efficiency, and environmental protection characteristics. The introduction of new clean energy sources in the materialization stage can reduce carbon emissions to a large extent (EN1). However, the development of new technologies and energy sources requires higher costs. Therefore, it is necessary to increase economy input in the research and development of new energy sources and technologies (EC1), and adequate financial security is a prerequisite for the progress of R&D efforts.

### 5.3. Suggestions for Carbon Reduction Paths during the Materialization Stage of Prefabricated Buildings

**Figure 7:**
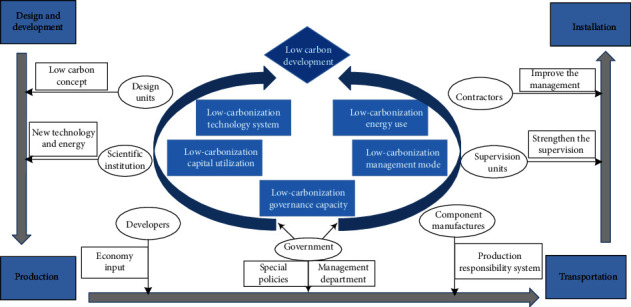
Carbon reduction path implementation for participants involved during the materialization stage of prefabricated buildings.

The study analyzed the carbon emission reduction paths during the materialization stage of prefabricated buildings by SEM and tested the proposed hypothesis. Ultimately, the paper clarified the interrelationships between the latent variables and identified PO2, MA2, MA3, MA6, TE1, TE3, EC1, and EN1 as the key paths to promote the low-carbon development of prefabricated buildings and proposed the following enhancement suggestions and conclusions (see Figure 7).

1. Strengthen policy guidance and promote low-carbonization governance ability. Government departments should improve low-carbon policies and give full play to the leading role. Especially in the production stage, the government should implement low-carbon for component manufacturers. On the basis of the original policy of tax reduction and financial subsidies for component manufacturers, special funds and special management departments can be set up. Special funds can alleviate the high cost of prefabricated buildings on the development of obstacles. Special management departments cannot only promote the effective use of the special funds but also can effectively supervise the component manufacturers. In addition, government departments should continue to improve low-carbon policies on technology, economy, and energy to ensure the government's leading position in the process of carbon emission reduction
2. Strengthen management supervision, and promote a low-carbonization management mode. Component manufacturers should cultivate low-carbon awareness among production personnel and implement the responsibility system for component production, which will improve the technical level of production personnel. At the same time, component manufacturers should strengthen the supervision and management of production personnel, so that they become high-level personnel in line with low-carbon development. At the component installation site, contractors and developers should consider the scale of the construction and the carbon emission of each engineering activity and formulate scientific and reasonable manpower, materials, and machinery scheduling plan. The supervision units shall ensure that all engineering activities can be carried out smoothly and efficiently to prevent unnecessary waste caused by site chaos and achieve low-carbon construction
3. Accelerate technology research and development, and promote low-carbonization technology systems. Technology is the core driving force of the low-carbon development of prefabricated buildings. Scientific research institutions and other units should accelerate the research and development and upgrading of low-carbon technologies. On this basis, design units should integrate low-carbon technologies into the design schemes according to low-carbon principles, throughout the whole process of the materialization stage of prefabricated buildings. In this way, resources and energy are used most efficiently with a minimal negative impact on the environment. At the same time, component manufacturers and government departments should improve the standardized production process of component manufacturing as soon as possible
4. Increase economy input, and promote low-carbonization capital utilization. Sufficient funds are an important guarantee for the research and development of new energy and new technologies, and the introduction of advanced energy-saving equipment also has high requirements for funds. Therefore, within the scope of the budget allowed, prefabricated building-related enterprises should increase economic input as much as possible. It is crucial to pay attention to the development of a plan for the use of funds to ensure that the funds invested are in place and prevent low-carbon development from being hindered by the lack of capital turnover. For government departments, some financial subsidy policies can be introduced
5. Optimize energy structure and promote low-carbonization energy use. The current energy consumption of the materialization stage of prefabricated buildings mainly comes from coal, oil, and other traditional fossil fuels, which produce a large number of carbon emissions, while the new clean energy such as solar energy and wind energy is environmentally friendly, clean, and pollution-free in line with the need for low-carbon development of prefabricated buildings. Scientific research institutions should conform to the trend of low-carbon development of the times and vigorously develop new clean energy. So clean energy can be more popular throughout the materialization stage, which can reduce the use of fossil fuels and further optimize the energy structure and reduce carbon emissions

## 6. Conclusion

Based on the literature review and the 3E system theory, the study constructed the scope of carbon emission reduction during the materialization stage of prefabricated buildings and used the questionnaire survey to collect the data and applied SEM to explore the impact of each dimension on carbon emission reduction during the materialization stage and the relationship between them. The hypotheses proposed in this study were tested, and the main conclusions are as follows:

1. “Management mode” has the strongest effects on carbon emission reduction during the materialization stage of prefabricated buildings, followed by “government policy,” “technology level,” and “economy input,” and “energy structure” has the weakest effects
2. The five carbon reduction impact dimensions identified in this study not only influence on carbon reduction but there are also relationships among the dimensions. Among them, “government policy” mainly affects “technology level,” “energy structure,” and “economy input” indirectly by influencing “management mode,” while “management mode” directly affects them. In addition, in the current low-carbon development of prefabricated buildings, the policies on “technology,” “economy,” and “energy” need to be improved
3. Compared with the design and transportation stage, the carbon emission reduction paths in the production and installation stage have a greater impact on the carbon emission reduction during the materialization stage of prefabricated buildings. In the implementation of carbon emission reduction paths in the future, all participants could focus on carbon emission reduction during the production and installation stages

The theoretical implications of this study are mainly reflected in the 3E system theory for the exploration of the carbon emission reduction path of prefabricated buildings. Through the realization of environmental friendliness, energy conservation, and low-carbon economy in the development of prefabricated buildings, the factors affecting the carbon emission reduction of prefabricated buildings are systematically explored, so that the 3E theory can be more effectively applied to the field of carbon emission reduction. In terms of practical significance, this study has a guiding role for participants during the materialization stage. For example, the study found that government policy guidance on technology, economy, and energy is insufficient. At the same time, component manufacturers, contractors, and other participants to improve the management level of technology, personnel, and energy is the key to reducing carbon emissions. Therefore, under such realistic circumstances, policymakers should take some effective measures, such as financial subsidies, regulatory agencies, and tax incentives, to ensure the smooth implementation of the carbon emission reduction path and achieve low-carbon governance capacity, management mode, and technology system, capital, and energy utilization.

## Figures and Tables

**Table 1:** List of influencing factors of carbon emission reduction during the materialization stage of prefabricated buildings.

Dimension	Substage	Descriptions	References
Government policy (PO)	Design and development stage	Government departments provide financial subsidies to departments that adopt low-carbon design solutions (PO1)	[3, 6, 18]
	Production stage	Tax breaks by government departments for component manufacturers that meet carbon emission standards (PO2)	
	Transportation stage	Government departments set carbon emission standards and implement incentives and penalties for different sizes of components and different modes of transportation (PO3)	
	Installation stage	Government departments set carbon emission standards and implement incentives and penalties for construction sites of different sizes (PO4)	

Management mode (MA)	Design and development stage	Design and development units cultivate low carbon and environmental awareness among professionals (MA1)	[6, 18, 19, 26]
	Production stage	The enterprises set consumption standards for the production of unit parts and implement them to individuals (MA2)	
		Component manufacturers require production workers to meet proficiency standards for operating low-carbon equipment and technology (MA3)	
	Transportation stage	Flexible selection of low-carbon and energy-saving loading and unloading solutions according to the size and other characteristics of the parts during transportation (MA4)	
		Fully consider the transportation route height limit, load limit, and other factors, and set a low-carbon feasible transportation plan for the transportation of components (MA5)	
	Installation stage	Contractors reduce unnecessary waste by optimizing the scheduling plan for manpower, materials, and machinery at the construction site (MA6)	
		Contractors reasonably arrange the stacking position of the components to avoid secondary transportation (MA7)	

Technology level (TE)	Design and development stage	Incorporate low-carbon technologies such as the Internet of Things (IoT) into design solutions (TE1)	[2, 3, 19]
	Production stage	Component manufacturers rely on technologies such as 3D printing and BIM to ensure the quality of components to reduce waste (TE2)	
		Component manufacturers use technologies such as robotic production to standardize the production process of components (TE3)	
	Installation stage	Contractors adopt advanced recycling technologies for waste components and materials to reduce construction waste generation (TE4)	
		Contractors apply low-carbon advanced component connection technology to reduce on-site pouring (TE5)	

Economy input (EC)	Design and development stage	Investing funds to accelerate new energy and technology research and development (EC1)	[3–5, 20]
	Production stage	Introduction of advanced energy-saving production equipment by component manufacturers (EC2)	
	Transportation stage	Component transport units adopt new transport methods that use clean energy (EC3)	
	Installation stage	Contractors introduce advanced energy-saving construction equipment (EC4)	

Energy structure (EN)	Design and development stage	Design and development units incorporate the use of clean energy as a power source into the design solution (EN1)	[6, 25, 29]
	Production stage	Component manufacturers optimize the structure of materials used for high carbon emission components (EN2)	
	Installation stage	Contractors choose to use of building materials with carbon sequestration effect (EN3)	

**Table 2:** Basic information of interviewees.

Variable	Description	Number	Percent	Variable	Description	Number	Percent
Gender	Male	158	72%	Age	18-35 years old	84	38%
	Female	62	28%		36-50 years old	97	44%
Educational background	College	23	11%		>50 years old	39	18%
	University	139	63%	Nature of work units	Component manufacturers	51	23%
	Postgraduate or higher	58	26%		Developers	37	17%
Working experience	<3 years	43	19%		Contractors	46	21%
	3-5 years	121	55%		Design units	33	15%
	6-10 years	48	22%		Supervisory units	24	11%
	>10years	8	4%		Scientific institutions and universities	29	13%

**Table 3:** The index system of the influencing factors of carbon emission reduction during the materialization stage of prefabricated buildings.

Dimension	Code	Mean value	Standard deviation	CITC	Cronbach's alpha	KMO
PO	PO1	3.65	0.989	0.681	0.864	0.861
	PO2	3.30	0.976	0.809		
	PO3	3.72	0.993	0.659		
	PO4	3.64	0.991	0.678		

MA	MA1	3.77	0.818	0.650	0.905	0.895
	MA2	3.72	0.811	0.708		
	MA3	3.55	0.851	0.750		
	MA4	3.90	0.787	0.629		
	MA5	3.90	0.808	0.659		
	MA6	3.26	1.065	0.736		
	MA7	3.81	0.792	0.689		

TE	TE1	3.70	0.807	0.638	0.875	0.843
	TE2	3.78	0.848	0.658		
	TE3	3.67	0.801	0.725		
	TE4	3.82	0.813	0.633		
	TE5	4.09	0.871	0.491		

EC	EC1	3.50	0.797	0.616	0.931	0.821
	EC2	3.97	0.779	0.572		
	EC3	3.90	0.791	0.579		
	EC4	3.84	0.800	0.590		

EN	EN1	3.31	1.005	0.696	0.832	0.712
	EN2	3.69	1.023	0.522		
	EN3	3.63	0.884	0.523		

**Table 4:** The fit indices of the first-order SEM.

Index	Estimation	Standard	Result
*χ* ^2^/df	2.209	(1, 3)	Accept
RMSEA	0.074	<0.08	Accept
RMR	0.044	<0.5	Accept
CFI	0.923	>0.9	Accept
TLI	0.912	>0.9	Accept
IFI	0.924	>0.9	Accept
PGFI	0.663	>0.5	Accept
PNFI	0.759	>0.5	Accept

**Table 5:** Standardized path coefficients and hypothesis testing of the first-order SEM.

Hypothesis	Relationship	Estimate	S.E.	C.R.	*P* value	Support
H1	PO⟶MA	0.717	0.079	8.677	^∗∗∗^	Yes
H2	PO⟶EC	0.135	0.127	1.487	0.137	No
H3	PO⟶EN	0.054	0.091	0.579	0.562	No
H4	PO⟶TE	0.028	0.089	0.314	0.753	No
H6	MA⟶EC	0.603	0.144	6.168	^∗∗∗^	Yes
H7	MA⟶EN	0.449	0.119	3.843	^∗∗∗^	Yes
H8	MA⟶TE	0.574	0.120	5.023	^∗∗∗^	Yes
H10	EC⟶TE	0.219	0.059	2.671	0.008^∗∗^	Yes
H11	EC⟶EN	0.268	0.061	3.033	0.002^∗∗^	Yes

^∗^ *p* < 0.05,  ^∗∗^*p* < 0.01, and^∗∗∗^*p* < 0.001.

**Table 6:** The fit indices of the second-order SEM.

Index	Estimation	Standard	Result
*χ* ^2^/df	2.178	(1, 3)	Accept
RMSEA	0.073	<0.08	Accept
RMR	0.044	<0.5	Accept
CFI	0.923	>0.9	Accept
TLI	0.914	>0.9	Accept
IFI	0.924	>0.9	Accept
PGFI	0.673	>0.5	Accept
PNFI	0.772	>0.5	Accept

**Table 7:** Standardized path coefficients and hypothesis testing of the second-order SEM.

Hypothesis	Relationship	Estimate	S.E.	C.R.	*P* value	Support
H5	PO⟵CA	0.746	0.048	9.735	^∗∗∗^	Yes
H9	MA⟵CA	0.923	0.047	11.458	^∗∗∗^	Yes
H12	EC⟵CA	0.775	0.052	9.761	^∗∗∗^	Yes
H13	EN⟵CA	0.738	0.059	11.425	^∗∗∗^	Yes
H14	TE⟵CA	0.801	0.053	8.543	^∗∗∗^	Yes

^∗^ *p* < 0.05,  ^∗∗^*p* < 0.01, and^∗∗∗^*p* < 0.001.

## Data Availability

Data for this study are available upon request to the corresponding author.

## Additional Points

*Limitation*. This study has some limitations that need to be addressed. The new round of promotion of prefabricated buildings in China has just begun, and practitioners have a low level of professionalism and incomplete understanding of the factors influencing carbon emission reduction, so a follow-up investigation is necessary. In view of the limitations, this study will revise and improve the carbon emission reduction factors in the future in the progress of the actual project, in order to ensure that the carbon emission reduction benefits of prefabricated buildings can be fully utilized.

## References

[1] Liu B., Zhang L., Sun J. D. (2020). Analysis and comparison of embodied energies in gross exports of the construction sector by means of their value-added origins. *Energy*.

[2] Du Q., Bao T. N., Li Y., Huang Y. D., Shao L. (2019). Impact of prefabrication technology on the cradle-to-site CO_2_ emissions of residential buildings. *Clean Technologies and Environmental Policy*.

[3] Lu W. S., Chen K., Xue F., Pan W. (2018). Searching for an optimal level of prefabrication in construction: an analytical framework. *Journal of Cleaner Production*.

[4] Wang H., Zhang Y. Q., Gao W. J., Kuroki S. (2020). Life cycle environmental and cost performance of prefabricated buildings. *Sustainability*.

[5] Hong J. K., Shen G. Q. P., Li Z. D., Zhang B. Y., Zhang W. Q. (2018). Barriers to promoting prefabricated construction in China: a cost-benefit analysis. *Journal of Cleaner Production*.

[6] Wu G. B., Yang R., Li L. (2019). Factors influencing the application of prefabricated construction in China: from perspectives of technology promotion and cleaner production. *Journal of Cleaner Production*.

[7] Ding Z. K., Liu S., Luo L. W., Liao L. H. (2020). A building information modeling-based carbon emission measurement system for prefabricated residential buildings during the materialization phase. *Journal of Cleaner Production*.

[8] Li X. J., Lai J. Y., Ma C. Y., Wang C. (2021). Using BIM to research carbon footprint during the materialization phase of prefabricated concrete buildings: a China study. *Journal of Cleaner Production*.

[9] Hao J. L., Cheng B. Q., Lu W. S. (2020). Carbon emission reduction in prefabrication construction during materialization stage: a BIM-based life-cycle assessment approach. *Science of the Total Environment*.

[10] Li X. J., Xie W. J., Jim C. Y., Feng F. (2021). Holistic LCA evaluation of the carbon footprint of prefabricated concrete stairs. *Journal of Cleaner Production*.

[11] Han Q. Y., Chang J. J., Liu G. W., Zhang H. (2022). The carbon emission assessment of a building with different prefabrication rates in the construction stage. *International Journal of Environmental Research and Public Health*.

[12] Wang S. Z., Sinha R. (2021). Life cycle assessment of different prefabricated rates for building construction. *Buildings*.

[13] Ji Y. B., Li K. J., Liu G. W., Shrestha A., Jing J. X. (2018). Comparing greenhouse gas emissions of precast in-situ and conventional construction methods. *Journal of Cleaner Production*.

[14] Navaratnam S., Ngo T., Gunawardena T., Henderson D. (2019). Performance review of prefabricated building systems and future research in Australia. *Buildings*.

[15] Teng Y., Li K. J., Pan W., Ng T. (2018). Reducing building life cycle carbon emissions through prefabrication: evidence from and gaps in empirical studies. *Building and Environment*.

[16] Teng Y., Pan W. (2020). Estimating and minimizing embodied carbon of prefabricated high-rise residential buildings considering parameter, scenario and model uncertainties. *Building and Environment*.

[17] Chang Y., Li X. D., Masanet E., Zhang L., Huang Z., Ries R. (2018). Unlocking the green opportunity for prefabricated buildings and construction in China. *Resources, Conservation and Recycling*.

[18] Sandanayake M., Luo W., Zhang G. M. (2019). Direct and indirect impact assessment in off-site construction--A case study in China. *Sustainable Cities and Society*.

[19] Luna-Tintos J. F., Cobreros C., Lopez-Escamilla A., Herrera-Limones R., Torres-Garcia M. (2020). Methodology to evaluate the embodied primary energy and CO_2_ production at each stage of the life cycle of prefabricated structural systems: the case of the solar decathlon competition. *Energies*.

[20] Xue X. L., Zhang X. L., Wang L., Skitmore M., Wang Q. (2018). Analyzing collaborative relationships among industrialized construction technology innovation organizations: a combined SNA and SEM approach. *Journal of Cleaner Production*.

[21] Ma X. J., Li Y. D., Zhang X. Y. (2018). Research on the ecological efficiency of the Yangtze River Delta region in China from the perspective of sustainable development of the economy-energy-environment (3E) system. *Environmental Science and Pollution Research*.

[22] Li X. J., Wang C., Kassem M. A., Bimenyimana S. (2022). Fairness theory-driven incentive model for prefabricated building development. *Arabian Journal for Science and Engineering*.

[23] Wang Y. N., Xue X. L., Yu T., Wang Y. W. (2021). Mapping the dynamics of China’ prefabricated building policies from 1956 to 2019: a bibliometric analysis. *Building Research and Information*.

[24] Du H., Han Q., Sun J., Wang C. C. (2022). Adoptions of prefabrication in residential sector in China: agent-based policy option exploration. *Engineering Construction and Architectural Management*.

[25] Luo L., Chen Y. Y. (2020). Carbon emission energy management analysis of LCA-based fabricated building construction. *Sustainable Computing: Informatics and Systems*.

[26] Wang H. N., Zhang H., Hou K. M., Yao G. (2021). Carbon emissions factor evaluation for assembled building during prefabricated component transportation phase. *Energy Exploration & Exploitation*.

[27] Yin S., Dong T., Li B. Z., Gao S. (2022). Developing a conceptual partner selection framework: digital green innovation management of prefabricated construction enterprises for sustainable urban development. *Buildings*.

[28] Dong L., Wang Y., Li H. X., Jiang B. Y., Al-Hussein M. (2018). Carbon reduction measures-based LCA of prefabricated temporary housing with renewable energy systems. *Sustainability*.

[29] Toosi H. A., Lavagna M., Leonforte F., Del Pero C., Aste N. (2020). Life cycle sustainability assessment in building energy retrofitting; a review. *Sustainable Cities and Society*.

[30] Mafimisebi I. B., Jones K., Sennaroglu B., Nwaubani S. (2018). A validated low carbon office building intervention model based on structural equation modelling. *Journal of Cleaner Production*.

[31] Hou J., Hou B. (2019). Farmers’ adoption of low-carbon agriculture in China: an extended theory of the planned behavior model. *Sustainability*.

[32] Yin J. H., Shi S. Q. (2019). Analysis of the mediating role of social network embeddedness on low-carbon household behaviour: evidence from China. *Journal of Cleaner Production*.

[33] Du Q., Pang Q. Y., Bao T. N., Guo X. Q., Deng Y. G. (2021). Critical factors influencing carbon emissions of prefabricated building supply chains in China. *Journal of Cleaner Production*.

[34] Liu J. K., Yi Y. Q., Wang X. T. (2020). Exploring factors influencing construction waste reduction: a structural equation modeling approach. *Journal of Cleaner Production*.

[35] Tan S. T., Ho W. S., Hashim H., Lee C. T., Taib M. R., Ho C. S. (2015). Energy, economic and environmental (3E) analysis of waste-to-energy (WTE) strategies for municipal solid waste (MSW) management in Malaysia. *Energy Conversion and Management*.

